# Supplementary material for: Magnesium(II) Porphyrazine with Thiophenylmethylene Groups-Synthesis, Electrochemical Characterization, UV–Visible Titration with Palladium Ions, and Density Functional Theory Calculations
Source: Molecules. 2024 Jul 30;29(15):3610. doi: 10.3390/molecules29153610 (PMC11314072; doi:10.3390/molecules29153610)
Supplement: Supplementary file 1 [file molecules-29-03610-s001.zip › molecules-3086480-supplementary.pdf]

Magnesium(II) Porphyrazine with Thiophenylmethylene Groups-Synthesis, Electrochemical Characterization, UV–Visible Titration with Palladium Ions, and Density Functional Theory Calculations

Wojciech Szczolko 1,*, Kyrylo Chornovolenko 1, Jacek Kujawski 2, Zbigniew Dutkiewicz 1 and Tomasz Koczorowski 1,*

1 Chair and Department of Chemical Technology of Drugs, Poznan University of Medical Sciences, Rokietnicka 3, 60-806 Poznan, Poland

2 Chair and Department of Organic Chemistry, Poznan University of Medical Sciences, Rokietnicka 3, 60-806 Poznan, Poland

* Correspondence: wszczolko@ump.edu.pl (W.S.); tkoczorowski@ump.edu.pl (T.K.)

Table of Contents

1. **NMR spectra (pages 3-8)**
2. **CV voltammograms of the electropolymerization of GC electrode modified with multi-walled carbon nanotubes and porphyrazine 7 in PBS. Scan rate 100 mV×s^{-1} (page 9)**
3. **Computational studies – cartesian coordinates of conformers I−VI (pages 9-27)**
4. **UV-vis spectra of pz 7 in DCM (page 28)**

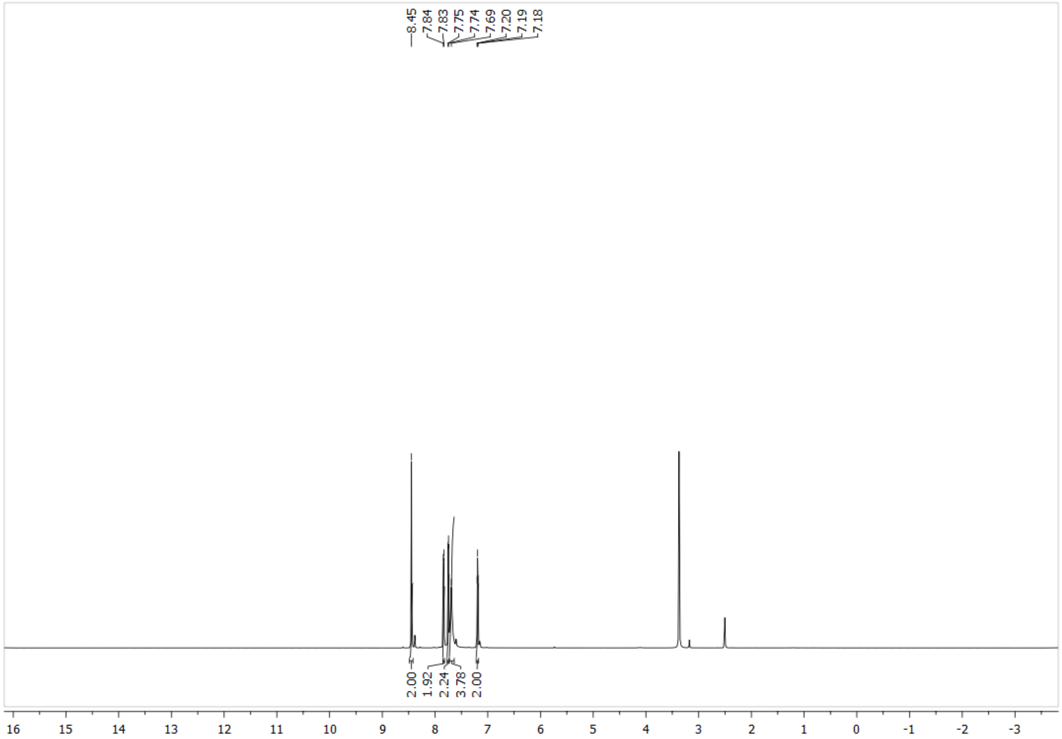

^{1}H NMR of 2-Amino-3-[(2-thiophenylmethylene)amino]-2-butene-1,4-dinitrile (**2**) in DMSO-*d*$_6$

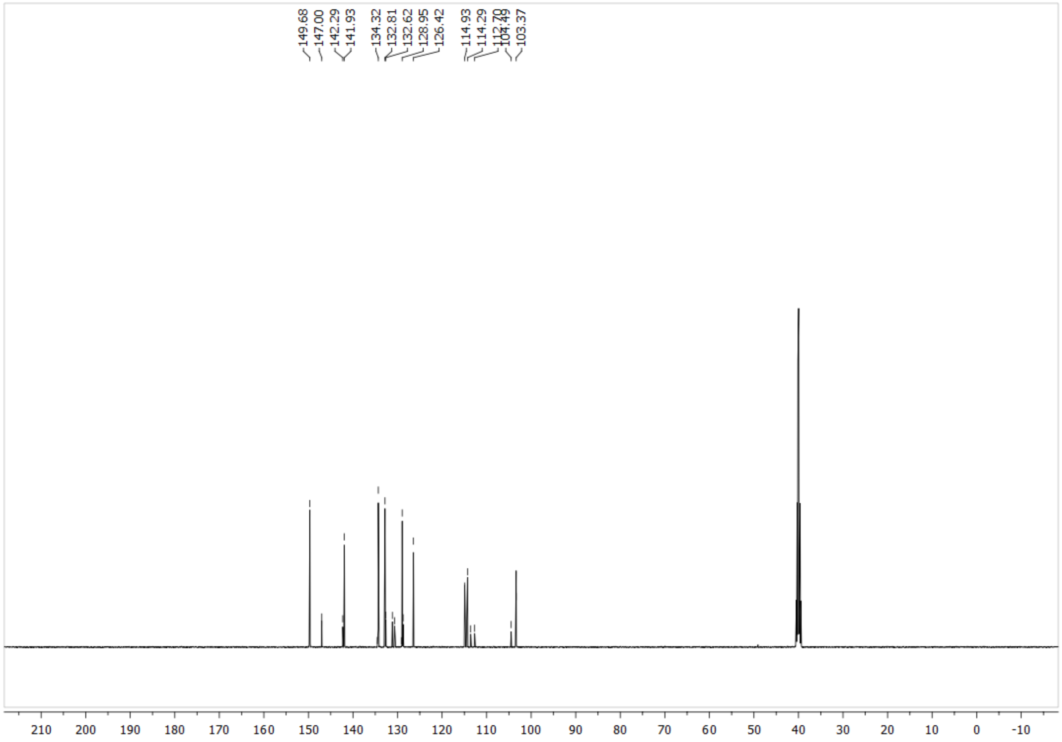

^{13}C NMR of 2-Amino-3-[(2-thiophenylmethylene)amino]-2-butene-1,4-dinitrile (**2**) in DMSO-*d*$_6$

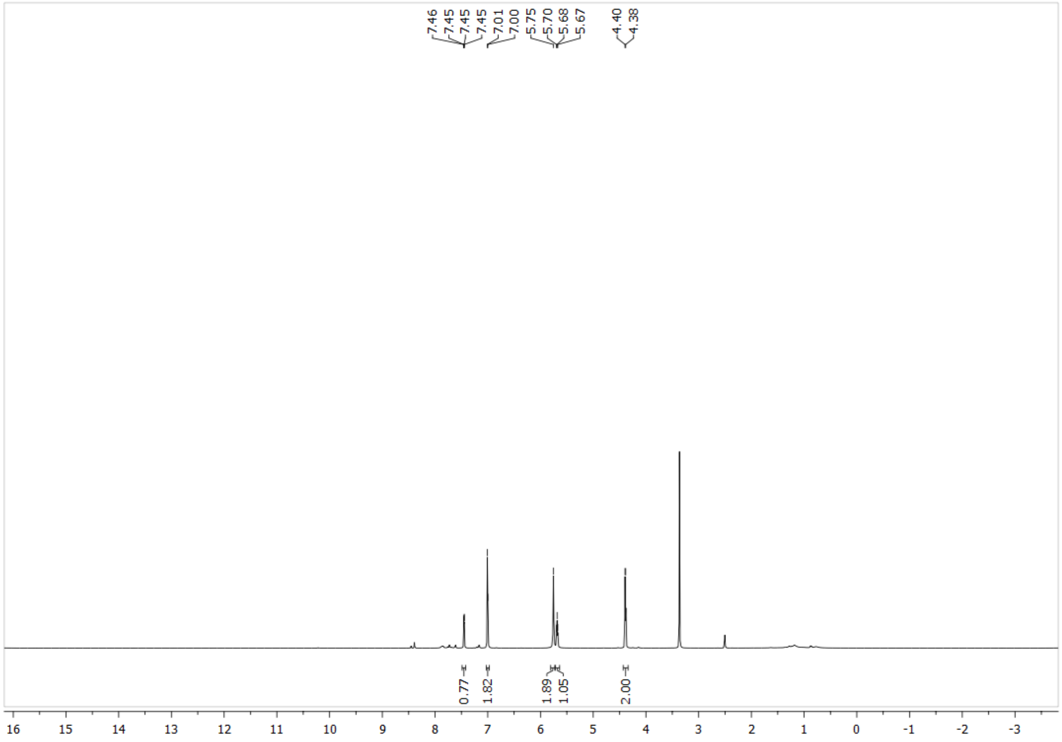

^{1}H NMR of 2-Amino-3-[(2-thiophenylmethyl)amino]-2-butene-1,4-dinitrile (**3**) in DMSO-d_6

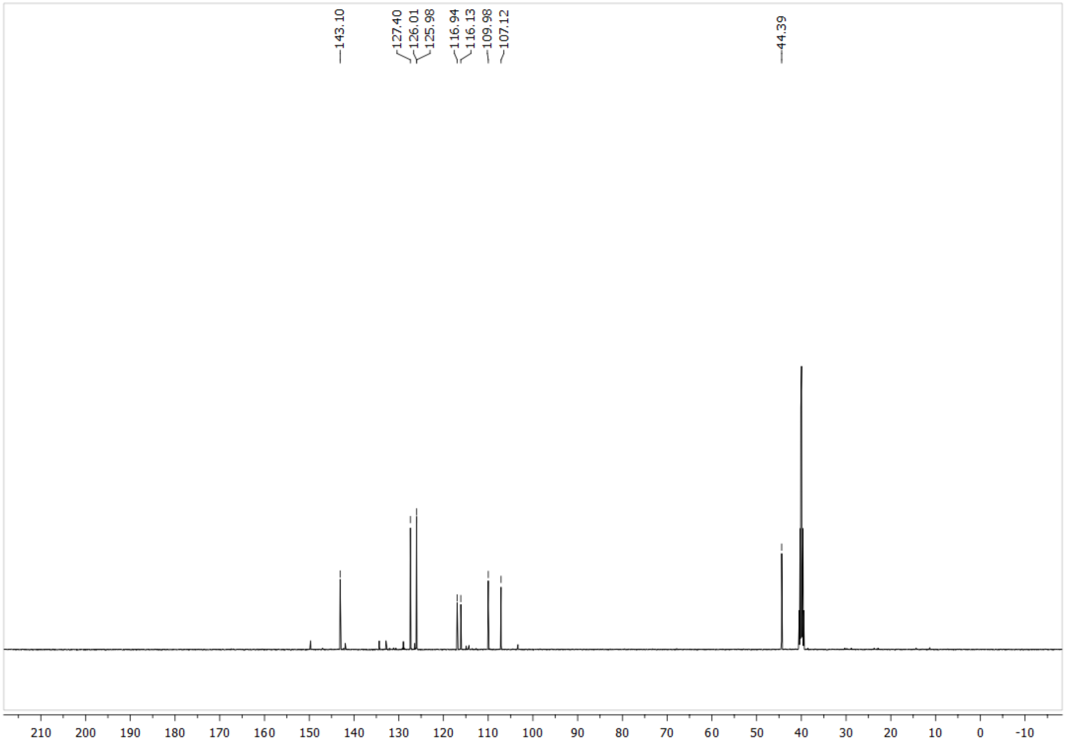

^{13}C NMR of 2-Amino-3-[(2-thiophenylmethyl)amino]-2-butene-1,4-dinitrile (**3**) in DMSO-d_6

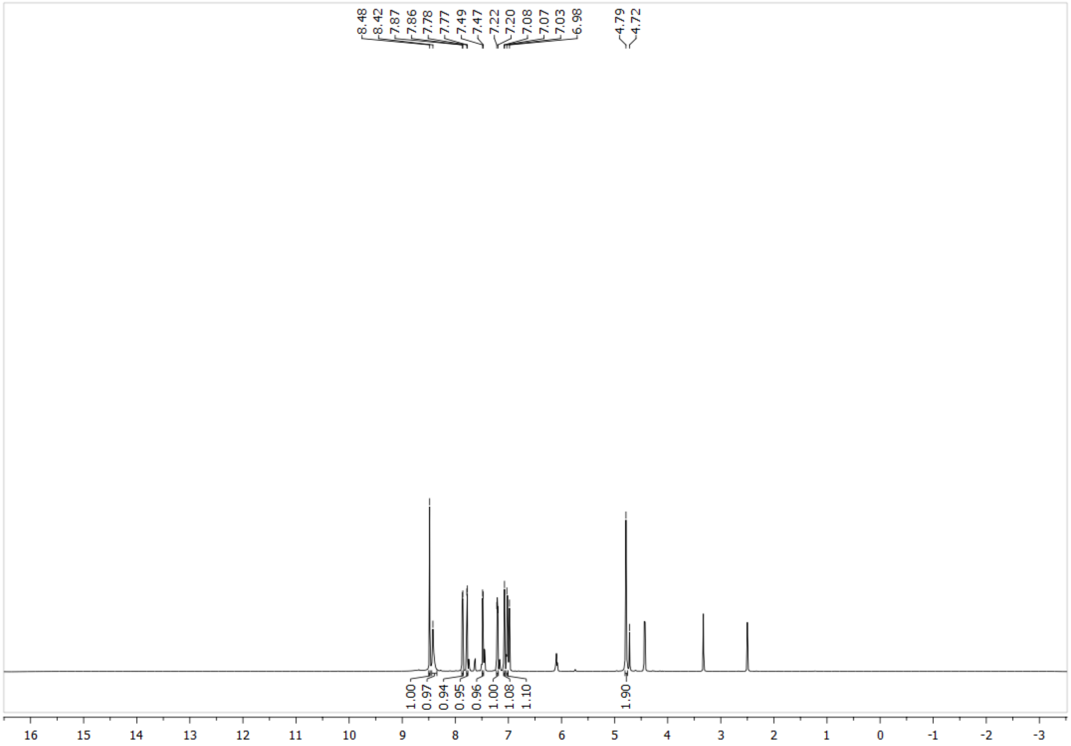

^{1}H NMR of 2-[(2-thiophenylmethylene)amino]-3-[(2-thiophenylmethyl)amino]-2-butene-1,4-dinitrile (**4**) in DMSO-d_6

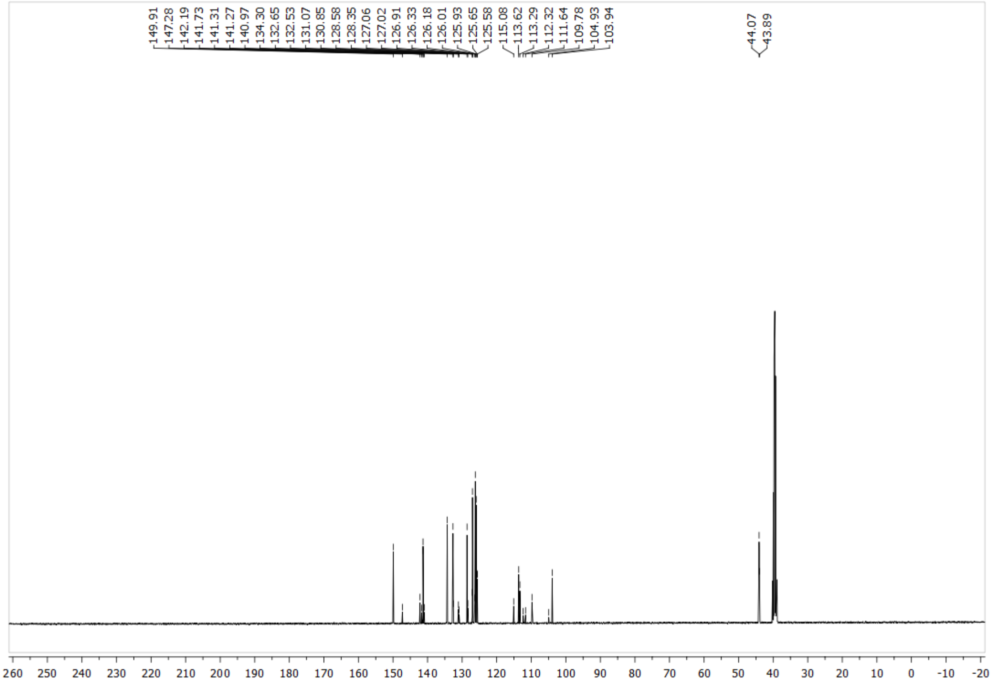

^{13}C NMR of 2-[(2-thiophenylmethylene)amino]-3-[(2-thiophenylmethyl)amino]-2-butene-1,4-dinitrile (**4**) in DMSO-d_6

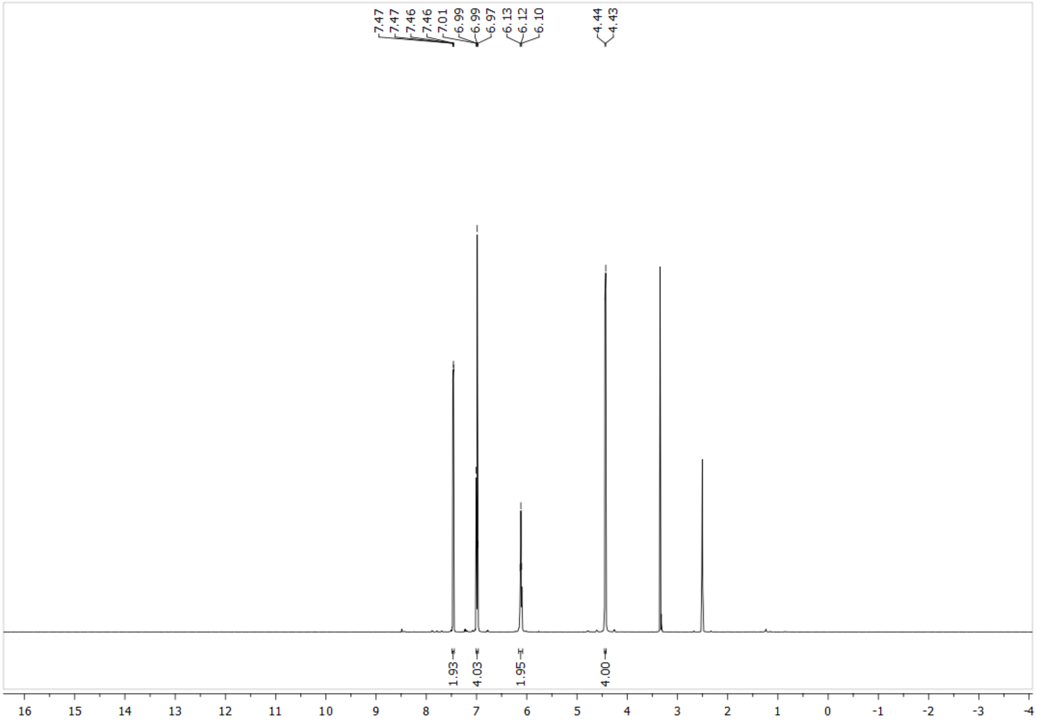

^{1}H NMR of 2,3-Bis-[(2-thiophenylmethyl)amino]-2(*Z*)-butene-1,4-dinitrile (**5**) in DMSO-d_6

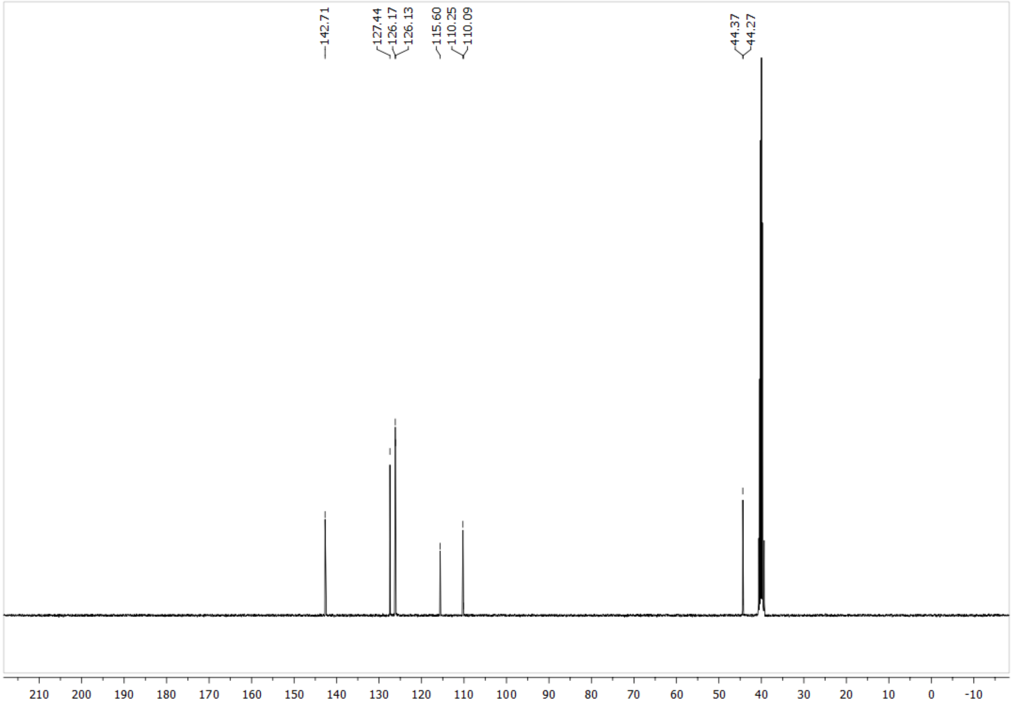

^{13}C NMR of 2,3-Bis-[(2-thiophenylmethyl)amino]-2(*Z*)-butene-1,4-dinitrile (**5**) in DMSO-d_6

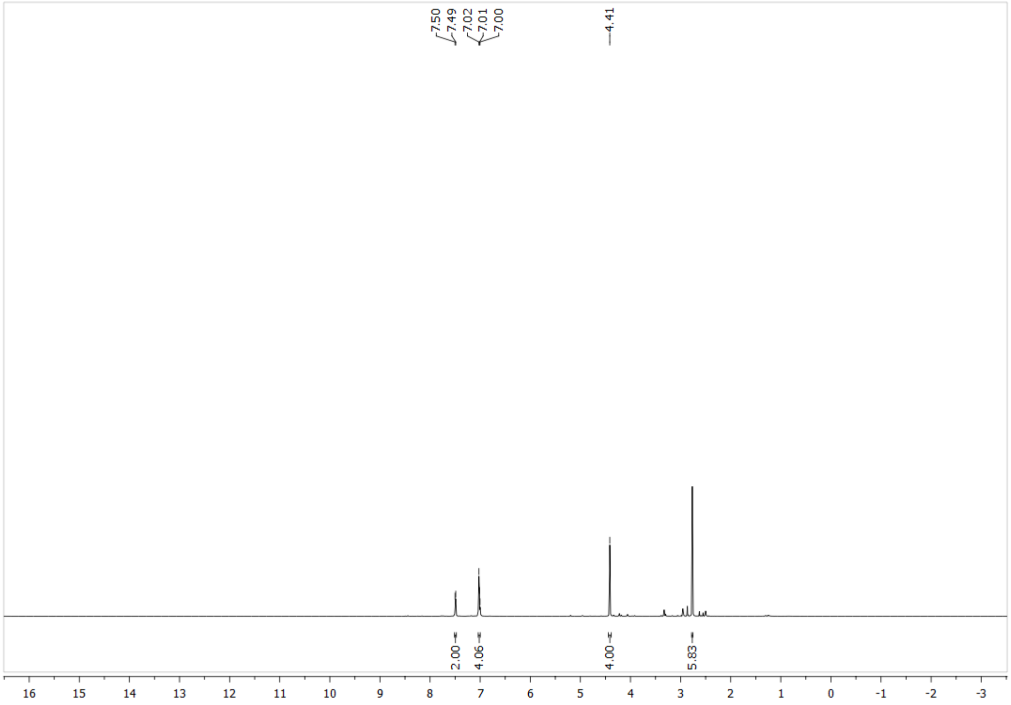

^{1}H NMR of 2,3-Bis-[methyl(2-thiophenylmethyl)amino]-2(*Z*)-butene-1,4-dinitrile (**6**) in DMSO-d_6

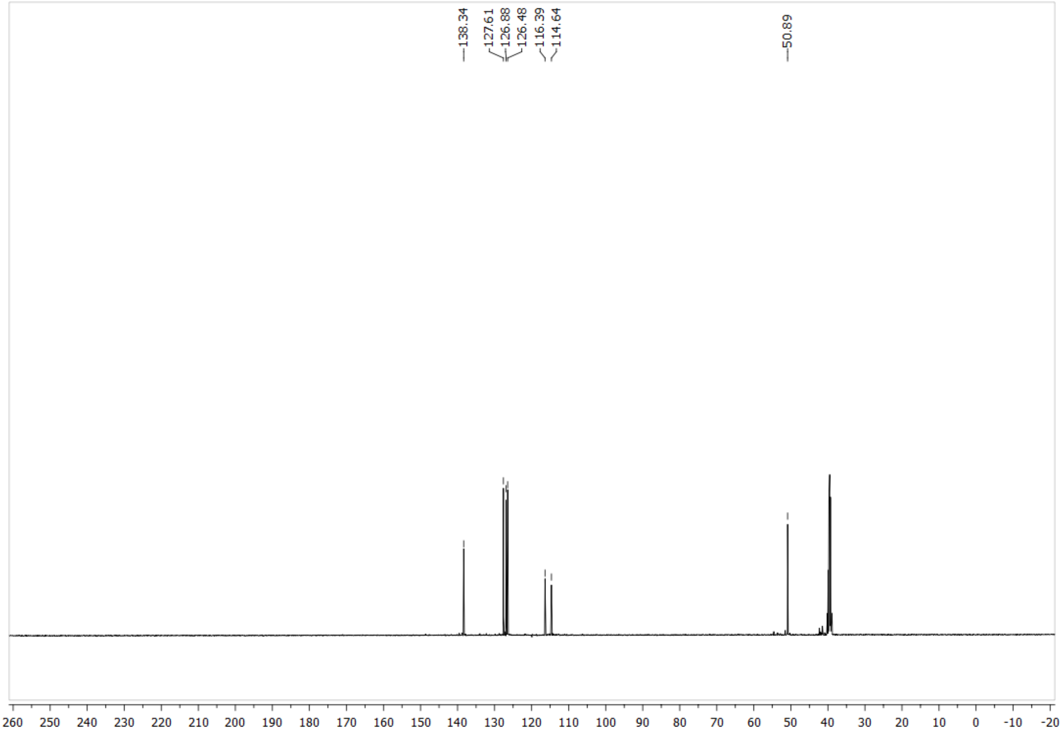

^{13}C NMR of 2,3-Bis-[methyl(2-thiophenylmethyl)amino]-2(*Z*)-butene-1,4-dinitrile (**6**) in DMSO-d_6

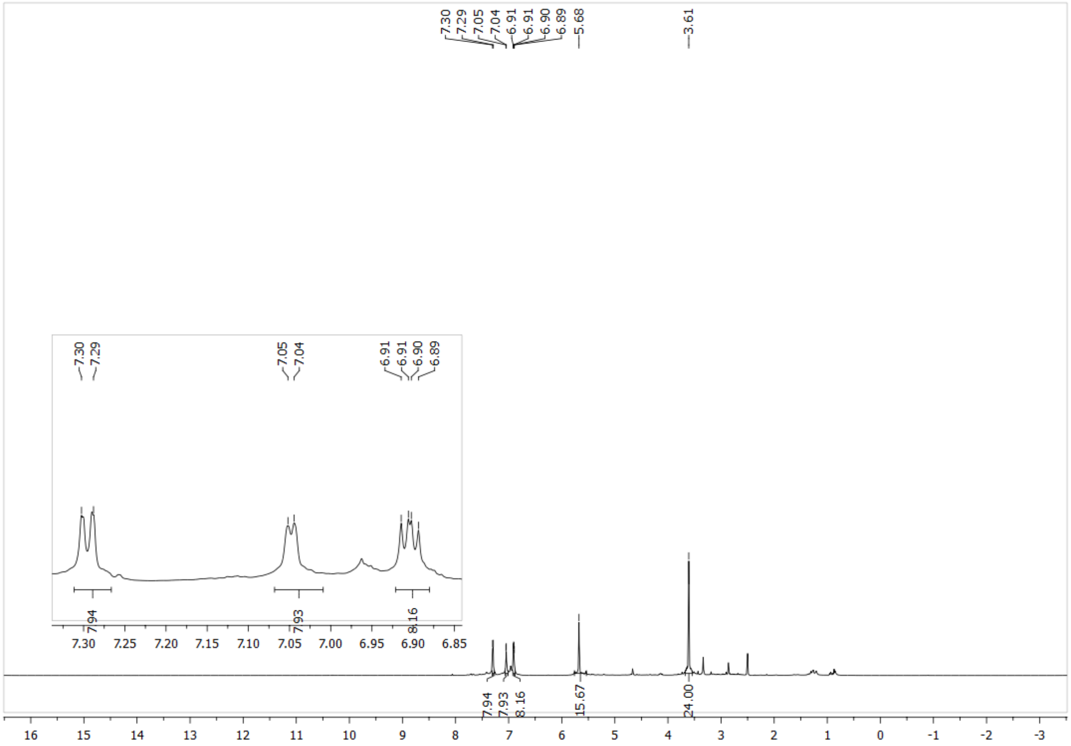

^{1}H NMR of [2,3,7,8,12,13,17,18-octakis-[methyl(2-thiophenylmethyl)amino]-porphyrazinato]magnesium (II) (**7**) in DMSO-d_6

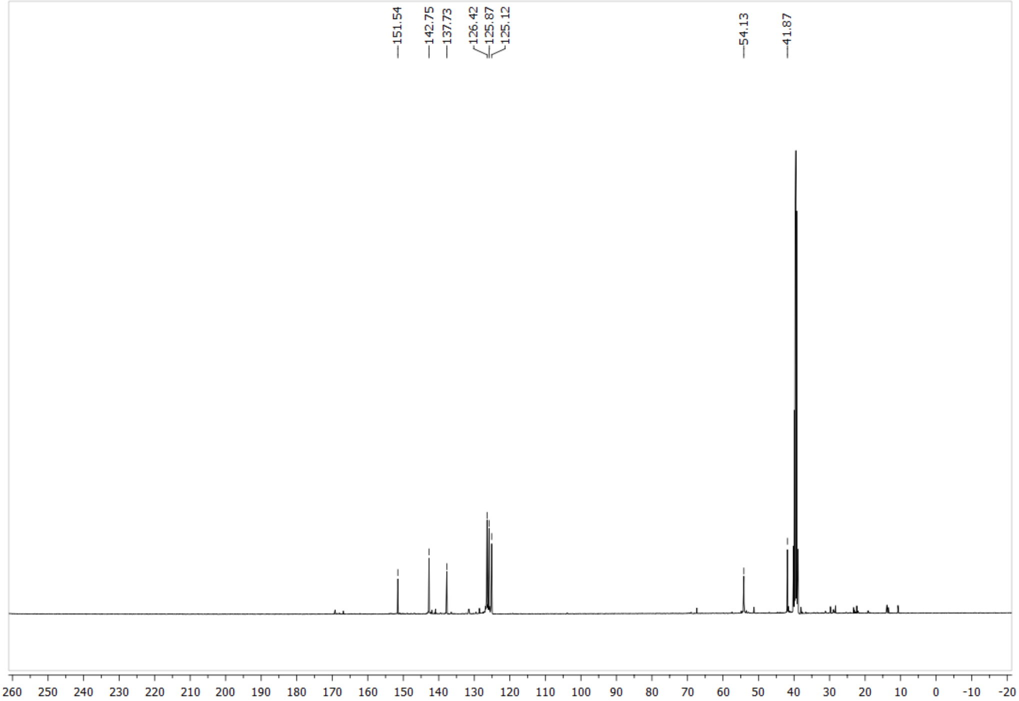

^{13}C NMR of 2,3,7,8,12,13,17,18-octakis-[methyl(2-thiophenylmethyl)amino]-porphyrazinato]magnesium (II) (7) in DMSO-d_6

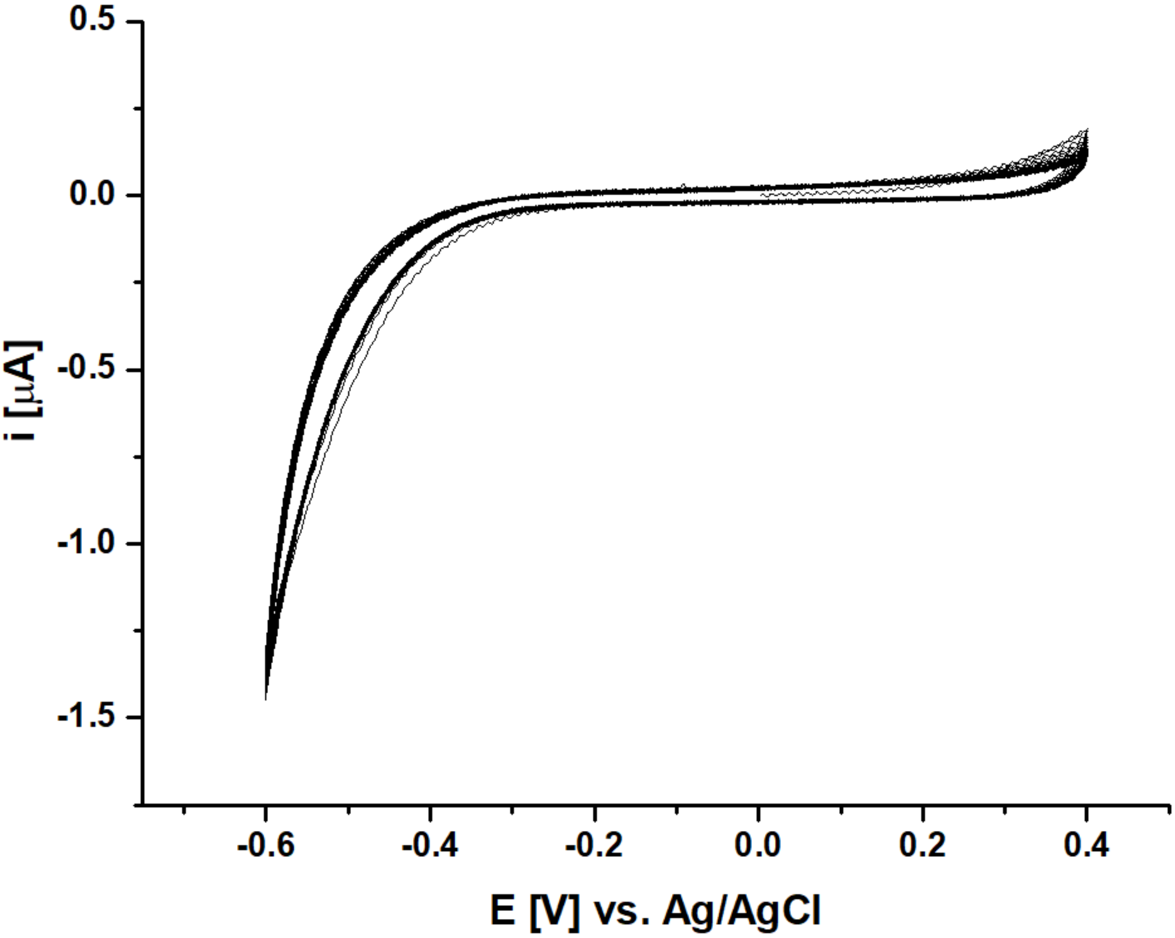

Cartesian coordinates (XYZ files) of conformers I-VI (compound 7; charge = 0, multiplicity = 1):

Conformer I

153

N	-0.757758000	-1.598823000	-0.240731000
N	-1.342142000	1.154491000	-0.474455000
N	1.414863000	1.760963000	-0.243478000
N	2.006796000	-0.995025000	-0.070039000
C	-2.103454000	-1.674517000	-0.494119000
C	-2.601964000	0.651882000	-0.636595000
C	2.777758000	1.831812000	-0.115706000
C	3.279568000	-0.493401000	-0.052540000
C	-2.516925000	-3.074769000	-0.526759000
C	-3.557135000	1.747239000	-0.765292000
C	3.186255000	3.236178000	-0.160165000
C	4.237668000	-1.635186000	0.012191000
C	-1.383888000	-3.838447000	-0.276390000

C	-2.829150000	2.928774000	-0.642286000
C	2.016347000	3.982597000	-0.360618000
C	3.444788000	-2.788623000	0.093327000
C	-0.255979000	-2.860701000	-0.134490000
C	-1.403798000	2.519843000	-0.500366000
C	0.918090000	3.026119000	-0.386973000
C	2.069481000	-2.356258000	0.027574000
N	-0.382106000	3.368572000	-0.482799000
N	1.017064000	-3.209048000	0.010192000
N	-2.947104000	-0.648914000	-0.663883000
N	3.627884000	0.793078000	-0.040836000
N	-4.971736000	1.630674000	-0.851781000
C	-5.457641000	0.752677000	-1.927937000
H	-4.823900000	0.938873000	-2.802633000
H	-5.346491000	-0.311170000	-1.677348000
C	-5.614766000	1.369685000	0.441468000
H	-5.290627000	2.120894000	1.165005000
H	-6.700415000	1.445028000	0.329705000
H	-5.375010000	0.371781000	0.843247000
C	-9.145537000	0.590423000	-2.839531000
C	-9.101764000	1.945321000	-3.008308000
S	-7.520880000	2.580221000	-2.658397000
C	-6.901194000	0.985053000	-2.281903000
C	-7.889735000	0.046300000	-2.422899000
H	-10.039100000	-0.003104000	-2.996913000
H	-9.900229000	2.614462000	-3.298103000
H	-7.721401000	-1.009312000	-2.237159000
H	-8.330380000	-6.655950000	0.504346000
C	-7.815249000	-5.706536000	0.454501000
C	-8.349042000	-4.450955000	0.384041000
H	-9.415155000	-4.253989000	0.378597000
H	-7.571412000	-2.370478000	0.248935000
C	-7.349079000	-3.430650000	0.309287000
C	-6.066375000	-3.913261000	0.327807000
S	-6.076795000	-5.662187000	0.431214000
C	-4.785691000	-3.126716000	0.321923000
H	-4.265453000	-3.252829000	1.277951000
H	-5.049405000	-2.061802000	0.238276000
H	-5.320459000	-3.810936000	-2.202482000
C	-4.348100000	-3.322642000	-2.091460000
H	-4.459651000	-2.257768000	-2.339172000
H	-3.658109000	-3.771161000	-2.811700000
N	-3.843743000	-3.550286000	-0.731689000
H	-2.683889000	-6.013808000	5.149481000
C	-2.210458000	-6.289235000	4.217257000
C	-2.028323000	-7.536344000	3.689154000
H	-2.357670000	-8.445497000	4.179251000
H	-1.126613000	-8.395979000	1.846943000
C	-1.362543000	-7.508183000	2.424318000
C	-1.038294000	-6.245769000	1.999454000
S	-1.562803000	-5.057381000	3.176271000
C	-0.342975000	-5.843456000	0.723116000
H	0.072198000	-6.749984000	0.268836000
H	0.484858000	-5.162961000	0.912728000

H	-1.784235000	-7.035743000	-1.065253000
C	-2.257085000	-6.080425000	-0.813663000
H	-3.078338000	-6.273568000	-0.115182000
H	-2.672657000	-5.644606000	-1.720298000
N	-1.225239000	-5.203579000	-0.268275000
H	7.421462000	-1.161045000	5.306068000
C	7.528586000	-1.440055000	4.266964000
C	8.670701000	-1.540021000	3.524026000
H	9.659868000	-1.341395000	3.920737000
H	9.193442000	-2.057043000	1.424411000
C	8.416778000	-1.926134000	2.170570000
C	7.089344000	-2.123519000	1.893229000
S	6.120005000	-1.823775000	3.322730000
C	6.469110000	-2.550768000	0.586631000
H	5.886164000	-3.465974000	0.686747000
H	7.286281000	-2.763855000	-0.112785000
C	6.303734000	-0.352159000	-0.503517000
N	5.600895000	-1.528524000	-0.009362000
H	-5.060641000	5.052015000	4.595300000
C	-4.699676000	5.373971000	3.628245000
C	-4.980482000	6.529615000	2.954889000
H	-5.635995000	7.301501000	3.341899000
H	-4.415115000	7.443298000	1.007775000
C	-4.319893000	6.604470000	1.689261000
C	-3.539693000	5.512646000	1.408018000
S	-3.614631000	4.360256000	2.725734000
C	-2.706837000	5.260557000	0.175504000
H	-1.689304000	4.972097000	0.434122000
H	-2.647765000	6.203863000	-0.379848000
H	-4.512458000	5.608913000	-1.635255000
C	-4.540632000	4.558404000	-1.327624000
H	-4.699954000	3.937789000	-2.208390000
H	-5.390454000	4.417020000	-0.650449000
N	-3.252721000	4.233464000	-0.725433000
H	-0.926076000	7.062863000	4.218724000
C	-0.110424000	6.640751000	3.648451000
C	0.675289000	5.563049000	3.951246000
H	0.560092000	4.978658000	4.857123000
H	2.384957000	4.492787000	3.017125000
C	1.663118000	5.299742000	2.953403000
C	1.625819000	6.172457000	1.894787000
S	0.348347000	7.348247000	2.130590000
C	2.489447000	6.157190000	0.657248000
H	3.461194000	5.733427000	0.926965000
H	2.662570000	7.176264000	0.295131000
H	1.255874000	6.983634000	-1.590254000
C	0.891385000	6.011841000	-1.235901000
H	-0.030040000	6.177229000	-0.665883000
N	1.953731000	5.372746000	-0.469547000
H	5.630040000	1.850922000	-5.289626000
C	5.796008000	2.454150000	-4.407766000
C	6.982927000	2.839603000	-3.849731000
H	7.949996000	2.567988000	-4.257674000
H	7.628997000	4.043131000	-2.094993000

C	6.807814000	3.636748000	-2.676192000
C	5.493777000	3.855613000	-2.347872000
S	4.442655000	3.064583000	-3.505116000
C	4.945279000	4.615695000	-1.166345000
H	5.744886000	5.256542000	-0.777680000
H	6.202082000	3.823891000	1.094848000
C	5.516770000	3.075577000	0.677668000
H	5.076064000	2.507223000	1.496398000
H	6.100314000	2.386648000	0.057492000
N	4.469629000	3.766698000	-0.060839000
H	6.225514000	-3.595162000	-4.972094000
C	5.403364000	-3.811642000	-4.303981000
C	4.062952000	-3.670519000	-4.529213000
H	3.649239000	-3.308563000	-5.463643000
H	2.187740000	-4.010357000	-3.380778000
C	3.271660000	-4.047520000	-3.399394000
C	4.009510000	-4.471030000	-2.325227000
S	5.719218000	-4.407133000	-2.702083000
C	3.504363000	-4.976023000	-1.005232000
H	2.412044000	-5.070466000	-1.081036000
H	4.142798000	-5.778437000	1.443283000
C	3.636324000	-4.807267000	1.428960000
H	4.042087000	-4.200102000	2.242731000
H	2.567749000	-4.971880000	1.626990000
N	3.901950000	-4.140307000	0.147228000
H	0.645699000	5.395549000	-2.101573000
H	4.118474000	5.264370000	-1.468057000
H	3.903661000	-5.979458000	-0.812309000
H	7.186065000	-0.688954000	-1.059853000
H	6.642954000	0.299729000	0.309579000
H	5.661029000	0.227406000	-1.160092000
Mg	0.328074000	0.085790000	-0.186200000

Conformer II

153

N	1.880635000	0.661316000	-1.546525000
C	3.005690000	-0.121707000	-1.466384000
C	4.185508000	0.720013000	-1.397569000
C	3.744287000	2.040674000	-1.517573000
C	2.261128000	1.969822000	-1.549517000
N	1.464705000	3.039220000	-1.493075000
H	7.110790000	6.873987000	2.280326000
C	6.560358000	6.111791000	1.746988000
C	5.318034000	5.610553000	2.015006000
H	4.704581000	5.947005000	2.843201000
H	3.960257000	4.087839000	1.131673000
C	4.913025000	4.601782000	1.087789000
C	5.847904000	4.343793000	0.120571000
S	7.253394000	5.359888000	0.342430000
C	5.799560000	3.327454000	-0.998401000
H	6.104398000	2.341472000	-0.641926000
H	6.513405000	3.617436000	-1.784303000

H	4.804913000	4.665921000	-3.039067000
C	4.015299000	4.370437000	-2.333607000
H	3.811687000	5.224615000	-1.679062000
N	4.465467000	3.206865000	-1.578573000
H	5.709979000	1.241820000	4.253902000
C	5.476538000	0.670096000	3.366585000
C	4.423759000	-0.174889000	3.151356000
H	3.659690000	-0.377763000	3.893253000
H	3.690501000	-1.444396000	1.477059000
C	4.442929000	-0.757110000	1.847277000
C	5.507583000	-0.356121000	1.082145000
S	6.509424000	0.764209000	1.976464000
C	5.838680000	-0.759439000	-0.325691000
H	6.912626000	-0.963574000	-0.411722000
H	5.739681000	-0.880209000	-3.120094000
C	6.144630000	0.019698000	-2.628943000
H	5.974092000	0.873043000	-3.290373000
H	7.226268000	-0.110322000	-2.510509000
N	5.536968000	0.289197000	-1.323452000
H	3.109100000	4.135579000	-2.885118000
H	5.308044000	-1.693404000	-0.544825000
N	-0.661310000	1.880622000	-1.546528000
C	0.121714000	3.005679000	-1.466384000
C	-0.720005000	4.185499000	-1.397600000
C	-2.040668000	3.744276000	-1.517578000
C	-1.969816000	2.261116000	-1.549516000
N	-3.039214000	1.464692000	-1.493070000
H	-6.873945000	7.110854000	2.280317000
C	-6.111761000	6.560406000	1.746978000
C	-5.610533000	5.318079000	2.015006000
H	-5.946979000	4.704641000	2.843214000
H	-4.087830000	3.960282000	1.131683000
C	-4.601771000	4.913051000	1.087787000
C	-4.343781000	5.847917000	0.120556000
S	-5.359890000	7.253402000	0.342383000
C	-3.327444000	5.799556000	-0.998417000
H	-2.341461000	6.104396000	-0.641948000
H	-3.617426000	6.513391000	-1.784327000
H	-4.665939000	4.804877000	-3.039049000
C	-4.370442000	4.015272000	-2.333583000
H	-5.224609000	3.811664000	-1.679023000
N	-3.206860000	4.465455000	-1.578573000
H	-1.241634000	5.710219000	4.253888000
C	-0.669972000	5.476712000	3.366548000
C	0.174944000	4.423877000	3.151313000
H	0.377823000	3.659831000	3.893232000
H	1.444362000	3.690514000	1.476995000
C	0.757112000	4.442977000	1.847210000
C	0.356152000	5.507635000	1.082064000
S	-0.764190000	6.509489000	1.976353000
C	0.759464000	5.838693000	-0.325782000
H	0.963603000	6.912636000	-0.411844000
H	0.880215000	5.739610000	-3.120180000
C	-0.019685000	6.144580000	-2.629037000

H	-0.873036000	5.974029000	-3.290457000
H	0.110343000	7.226222000	-2.510637000
N	-0.289181000	5.536960000	-1.323526000
H	-4.135588000	3.109068000	-2.885088000
H	1.693425000	5.308047000	-0.544911000
N	-1.880617000	-0.661323000	-1.546516000
C	-3.005672000	0.121702000	-1.466372000
C	-4.185492000	-0.720017000	-1.397578000
C	-3.744270000	-2.040677000	-1.517571000
C	-2.261110000	-1.969829000	-1.549496000
N	-1.464686000	-3.039226000	-1.493050000
H	-7.111029000	-6.873901000	2.280233000
C	-6.560552000	-6.111727000	1.746909000
C	-5.318226000	-5.610518000	2.014975000
H	-4.704820000	-5.946973000	2.843202000
H	-3.960382000	-4.087834000	1.131696000
C	-4.913156000	-4.601761000	1.087769000
C	-5.847988000	-4.343758000	0.120510000
S	-7.253492000	-5.359851000	0.342289000
C	-5.799578000	-3.327420000	-0.998461000
H	-6.104407000	-2.341431000	-0.641997000
H	-6.513401000	-3.617385000	-1.784388000
H	-4.804898000	-4.665919000	-3.039076000
C	-4.015291000	-4.370440000	-2.333605000
H	-3.811697000	-5.224618000	-1.679054000
N	-4.465462000	-3.206859000	-1.578586000
H	-5.710164000	-1.241780000	4.253900000
C	-5.476671000	-0.670092000	3.366574000
C	-4.423852000	0.174845000	3.151354000
H	-3.659804000	0.377718000	3.893274000
H	-3.690515000	1.444313000	1.477062000
C	-4.442967000	0.757044000	1.847265000
C	-5.507622000	0.356085000	1.082115000
S	-6.509457000	-0.764289000	1.976383000
C	-5.838691000	0.759422000	-0.325722000
H	-6.912636000	0.963555000	-0.411774000
H	-5.739626000	0.880226000	-3.120120000
C	-6.144586000	-0.019687000	-2.628990000
H	-5.974032000	-0.873024000	-3.290426000
H	-7.226228000	0.110330000	-2.510580000
N	-5.536954000	-0.289202000	-1.323488000
H	-3.109083000	-4.135592000	-2.885105000
H	-5.308054000	1.693393000	-0.544836000
N	0.661329000	-1.880629000	-1.546508000
C	-0.121696000	-3.005683000	-1.466359000
C	0.720025000	-4.185500000	-1.397542000
C	2.040684000	-3.744281000	-1.517564000
C	1.969834000	-2.261122000	-1.549498000
N	3.039232000	-1.464697000	-1.493072000
H	6.874144000	-7.110927000	2.280060000
C	6.111931000	-6.560471000	1.746771000
C	5.610728000	-5.318143000	2.014837000
H	5.947234000	-4.704711000	2.843026000
H	4.087996000	-3.960322000	1.131606000

C	4.601917000	-4.913098000	1.087679000
C	4.343862000	-5.847955000	0.120457000
S	5.359945000	-7.253467000	0.342237000
C	3.327469000	-5.799571000	-0.998464000
H	2.341500000	-6.104404000	-0.641949000
H	3.617404000	-6.513404000	-1.784394000
H	4.665880000	-4.804904000	-3.039143000
C	4.370422000	-4.015295000	-2.333666000
H	5.224622000	-3.811694000	-1.679146000
N	3.206871000	-4.465465000	-1.578602000
H	1.241705000	-5.710061000	4.253985000
C	0.670026000	-5.476583000	3.366648000
C	-0.174899000	-4.423761000	3.151396000
H	-0.377768000	-3.659692000	3.893295000
H	-1.444331000	-3.690442000	1.477069000
C	-0.757082000	-4.442898000	1.847301000
C	-0.356121000	-5.507569000	1.082176000
S	0.764188000	-6.509428000	1.976499000
C	-0.759440000	-5.838658000	-0.325662000
H	-0.963592000	-6.912600000	-0.411695000
H	-0.880215000	-5.739657000	-3.120057000
C	0.019689000	-6.144616000	-2.628913000
H	0.873033000	-5.974085000	-3.290346000
H	-0.110340000	-7.226253000	-2.510479000
N	0.289202000	-5.536958000	-1.323422000
H	4.135552000	-3.109090000	-2.885161000
H	-1.693395000	-5.308008000	-0.544799000
Mg	0.000009000	-0.000003000	-1.559694000

Conformer III

153

N	3.559386000	6.451828000	26.486845000
N	3.501292000	4.298942000	28.297521000
N	3.238308000	2.488722000	26.136414000
N	3.322500000	4.640733000	24.326901000
C	3.655295000	7.146333000	27.654577000
C	3.716505000	5.325844000	29.176221000
C	3.097044000	1.798132000	24.970044000
C	3.237398000	3.602857000	23.436918000
C	3.739939000	8.600518000	27.345997000
C	3.839160000	4.783238000	30.530832000
C	2.983326000	0.346889000	25.283200000
C	3.218876000	4.140367000	22.075199000
C	3.787051000	8.686059000	25.951109000
C	3.625120000	3.408028000	30.428184000
C	3.133923000	0.246966000	26.668793000
C	3.251537000	5.531615000	22.197023000
C	3.625325000	7.339653000	25.440949000
C	3.454025000	3.121581000	28.998991000
C	3.264826000	1.595465000	27.180284000
C	3.347184000	5.820985000	23.631727000
N	3.345562000	1.892646000	28.486808000

N	3.500943000	7.047223000	24.137125000
N	3.753248000	6.634224000	28.884282000
N	3.120668000	2.301307000	23.732876000
N	4.076564000	5.478249000	31.718598000
C	4.806296000	6.746847000	31.696410000
H	5.415902000	6.770232000	30.791196000
H	4.123059000	7.605723000	31.629982000
C	3.035089000	5.393503000	32.742885000
H	2.659496000	4.370970000	32.799067000
H	3.453527000	5.669307000	33.714193000
H	2.192306000	6.068370000	32.520185000
C	6.608168000	7.888565000	34.846355000
C	7.403384000	6.786109000	34.712186000
S	6.952274000	5.838086000	33.329628000
C	5.662587000	6.947440000	32.919295000
C	5.614809000	7.976401000	33.822651000
H	6.717670000	8.610941000	35.647270000
H	8.218540000	6.464305000	35.345272000
H	4.888122000	8.778999000	33.753360000
H	9.039350000	10.671843000	29.883876000
C	8.022044000	10.968078000	29.669243000
C	7.306195000	11.999449000	30.207427000
H	7.705817000	12.680642000	30.950044000
H	5.250159000	12.819803000	29.994796000
C	5.975576000	12.071779000	29.692036000
C	5.691338000	11.103547000	28.764671000
S	7.082565000	10.071028000	28.517148000
C	4.406341000	10.894500000	28.005599000
H	4.551686000	10.969289000	26.926892000
H	3.716716000	11.698618000	28.296860000
H	2.573279000	10.331265000	29.823953000
C	3.221442000	9.467743000	29.619580000
H	2.643366000	8.551910000	29.702537000
H	4.008834000	9.450860000	30.382247000
N	3.771766000	9.603114000	28.279622000
H	6.225297000	14.632660000	23.430321000
C	5.968913000	13.592891000	23.281569000
C	5.844497000	12.908467000	22.105993000
H	6.007067000	13.358241000	21.132893000
H	5.327658000	10.831009000	21.495920000
C	5.467348000	11.543784000	22.301405000
C	5.312397000	11.199116000	23.618373000
S	5.631143000	12.577731000	24.649759000
C	4.984257000	9.843647000	24.178918000
H	5.857783000	9.431245000	24.696976000
H	4.772428000	9.170328000	23.337244000
H	2.725815000	11.287124000	24.132852000
C	2.598813000	10.316589000	24.620155000
H	2.179331000	9.607096000	23.891811000
H	1.885077000	10.433300000	25.440060000
N	3.886491000	9.870481000	25.166358000
H	-2.248309000	2.091743000	22.162009000
C	-1.389112000	2.195308000	21.513969000
C	-1.003651000	1.388987000	20.479399000

H	-1.551536000	0.504320000	20.175236000
H	0.654864000	1.343062000	19.000826000
C	0.193016000	1.843134000	19.845892000
C	0.709314000	2.988224000	20.398356000
S	-0.293855000	3.524152000	21.727134000
C	1.971112000	3.718226000	20.012486000
H	1.809764000	4.800056000	20.011133000
H	2.232252000	3.422040000	18.989230000
C	3.682437000	2.096340000	20.750284000
N	3.133440000	3.439444000	20.871991000
H	3.931433000	1.119036000	36.964736000
C	4.308570000	1.135917000	35.951588000
C	5.137117000	0.239210000	35.338778000
H	5.535648000	-0.640609000	35.830942000
H	6.044853000	0.000390000	33.323396000
C	5.411552000	0.586629000	33.980759000
C	4.798559000	1.741880000	33.570858000
S	3.857586000	2.424886000	34.879752000
C	4.907048000	2.403338000	32.226452000
H	5.644201000	1.828192000	31.640808000
H	5.294757000	3.420108000	32.334292000
H	2.645166000	0.876555000	32.355226000
C	2.905351000	1.240585000	31.357224000
H	1.988209000	1.413768000	30.794008000
H	3.481183000	0.462016000	30.840549000
N	3.631060000	2.499191000	31.491288000
H	1.024377000	-6.018259000	28.596633000
C	1.141542000	-4.966459000	28.817849000
C	1.034150000	-4.336046000	30.024745000
H	0.796936000	-4.850048000	30.949299000
H	1.242355000	-2.255522000	30.782031000
C	1.279334000	-2.930875000	29.933894000
C	1.565554000	-2.502623000	28.664435000
S	1.544541000	-3.851230000	27.548449000
C	1.820339000	-1.096952000	28.197373000
H	1.789805000	-0.438466000	29.079222000
H	1.014137000	-0.771629000	27.530620000
H	4.193081000	-2.185558000	28.718490000
C	4.283700000	-1.205296000	28.242256000
H	4.454194000	-0.445510000	29.015987000
N	3.078660000	-0.947466000	27.442818000
H	7.987207000	-2.038745000	22.703187000
C	6.940864000	-2.242989000	22.883011000
C	6.115031000	-3.099653000	22.211802000
H	6.438562000	-3.709285000	21.375758000
H	3.980409000	-3.710721000	22.334083000
C	4.783499000	-3.097735000	22.729913000
C	4.605892000	-2.247827000	23.790521000
S	6.103940000	-1.423267000	24.164551000
C	3.345071000	-1.995530000	24.578346000
H	2.601419000	-2.737054000	24.260252000
H	1.495722000	-1.255672000	22.849879000
C	2.195606000	-0.435963000	23.058484000
H	1.659544000	0.508789000	23.032744000

H	2.951362000	-0.425605000	22.264565000
N	2.782357000	-0.656640000	24.371343000
H	8.728747000	7.708445000	21.712062000
C	7.786521000	7.660220000	21.184156000
C	7.280559000	8.540561000	20.269036000
H	7.802061000	9.435746000	19.950223000
H	5.430438000	8.710771000	19.047288000
C	5.994383000	8.149712000	19.785650000
C	5.532278000	6.978722000	20.332155000
S	6.699624000	6.337120000	21.464797000
C	4.214637000	6.293868000	20.071824000
H	3.791514000	6.725887000	19.156426000
H	2.287429000	8.135783000	20.373793000
C	2.699600000	7.788493000	21.330019000
H	1.900447000	7.772679000	22.070731000
H	3.456411000	8.509009000	21.660613000
N	3.219373000	6.441480000	21.144752000
H	5.153051000	-1.226868000	27.579001000
H	3.498641000	-2.131214000	25.649490000
H	4.356128000	5.223708000	19.896963000
H	4.011041000	1.945159000	19.713698000
H	2.961867000	1.311199000	21.001705000
H	4.543641000	1.990850000	21.409512000
Mg	3.396442000	4.469904000	26.312548000

Conformer IV

153

N	4.340432000	6.142436000	26.363241000
N	4.425050000	4.080256000	28.284749000
N	4.037610000	2.165222000	26.245853000
N	4.017816000	4.220545000	24.317736000
C	4.638906000	6.884925000	27.478838000
C	4.640063000	5.150347000	29.108545000
C	3.833303000	1.411500000	25.118268000
C	3.887344000	3.138137000	23.488065000
C	4.772119000	8.289586000	27.110888000
C	4.698376000	4.704079000	30.493735000
C	3.760493000	0.002194000	25.496433000
C	3.814760000	3.624100000	22.081626000
C	4.532015000	8.377592000	25.745429000
C	4.469911000	3.329348000	30.480757000
C	3.974028000	-0.048451000	26.880395000
C	3.828370000	5.022957000	22.165652000
C	4.285713000	6.972854000	25.282988000
C	4.340998000	2.947178000	29.048238000
C	4.116005000	1.329185000	27.325186000
C	3.971764000	5.357447000	23.560969000
N	4.232758000	1.697815000	28.615707000
N	4.094487000	6.626790000	24.016666000
N	4.764532000	6.435528000	28.731897000
N	3.776907000	1.864074000	23.855303000

N	4.808063000	5.528605000	31.645903000
C	5.926747000	6.482810000	31.626629000
H	6.783479000	5.960795000	31.184704000
H	5.722325000	7.349738000	30.983734000
C	3.535265000	6.135699000	32.052196000
H	2.778278000	5.355020000	32.154097000
H	3.659890000	6.624333000	33.022883000
H	3.170601000	6.882490000	31.327766000
C	6.907073000	8.464818000	34.716554000
C	6.992106000	7.273194000	35.378583000
S	6.577020000	5.938312000	34.346846000
C	6.288797000	7.005226000	32.989707000
C	6.503067000	8.308798000	33.354230000
H	7.115998000	9.422679000	35.179482000
H	7.254039000	7.096392000	36.412582000
H	6.379461000	9.134774000	32.661465000
H	4.306576000	14.387281000	30.321577000
C	4.231897000	13.308960000	30.347181000
C	4.164260000	12.489140000	31.437609000
H	4.169252000	12.850807000	32.459646000
H	4.033602000	10.298501000	31.802932000
C	4.095443000	11.106483000	31.081416000
C	4.105629000	10.885645000	29.728615000
S	4.211404000	12.403710000	28.864541000
C	3.990668000	9.571715000	29.009285000
H	3.037563000	9.521634000	28.470707000
H	3.973010000	8.774208000	29.767198000
H	6.576397000	10.210369000	29.173997000
C	6.403248000	9.310342000	28.577604000
H	6.574074000	8.427738000	29.209099000
H	7.135547000	9.294683000	27.764839000
N	5.051819000	9.361026000	28.006227000
H	-0.680466000	11.381389000	25.545466000
C	0.257240000	11.276650000	25.017716000
C	0.865444000	12.168968000	24.180938000
H	0.447173000	13.138501000	23.935676000
H	2.743404000	12.246295000	22.993958000
C	2.109972000	11.685325000	23.673339000
C	2.438138000	10.431839000	24.120458000
S	1.194636000	9.825889000	25.193597000
C	3.664755000	9.619052000	23.791275000
H	4.190615000	10.127145000	22.973911000
H	3.406614000	8.620929000	23.442390000
H	5.553264000	11.255789000	24.460930000
C	5.225032000	10.710956000	25.353511000
H	4.545853000	11.355860000	25.921589000
H	6.092089000	10.496484000	25.974457000
N	4.606339000	9.466050000	24.910910000
H	-1.626585000	1.894385000	19.577735000
C	-0.577716000	2.020574000	19.347933000
C	0.135495000	1.486738000	18.312459000
H	-0.299272000	0.841445000	17.557580000
H	2.242829000	1.546062000	17.604569000
C	1.512737000	1.866754000	18.340387000

C	1.836674000	2.688449000	19.388046000
S	0.423387000	3.001792000	20.373043000
C	3.178017000	3.298036000	19.708422000
H	3.136635000	4.386605000	19.746627000
H	3.866090000	3.033530000	18.895782000
C	4.164726000	1.431195000	20.966609000
N	3.747144000	2.826109000	20.974403000
H	-0.816778000	2.825514000	33.600041000
C	0.115523000	2.312684000	33.408120000
C	0.721974000	1.344043000	34.157327000
H	0.307499000	0.955688000	35.080762000
H	2.592369000	0.147807000	34.038118000
C	1.961345000	0.910556000	33.593965000
C	2.286997000	1.544294000	32.422861000
S	1.047233000	2.703709000	31.996720000
C	3.499503000	1.318347000	31.553349000
H	3.215260000	1.093032000	30.526708000
H	4.021053000	0.435525000	31.943785000
H	5.281274000	1.845557000	33.329506000
C	5.052966000	2.781061000	32.807452000
H	5.981211000	3.325266000	32.639239000
H	4.411262000	3.390890000	33.452851000
N	4.443812000	2.442087000	31.527238000
H	-0.490030000	-0.797397000	31.212655000
C	0.024806000	-0.895175000	30.267363000
C	-0.261897000	-0.291719000	29.074552000
H	-1.098025000	0.384170000	28.936342000
H	0.591903000	-0.275815000	27.022494000
C	0.653304000	-0.648943000	28.038888000
C	1.634012000	-1.517930000	28.445716000
S	1.425234000	-1.910676000	30.139117000
C	2.789628000	-2.050928000	27.636126000
H	2.467697000	-2.146780000	26.594932000
H	3.075152000	-3.049585000	27.986299000
H	5.064011000	-2.307802000	29.070708000
C	4.786816000	-1.263366000	28.879068000
H	4.259718000	-0.878759000	29.759151000
N	4.002429000	-1.212936000	27.651750000
H	8.799201000	-0.533477000	22.572606000
C	7.871173000	-1.053513000	22.765558000
C	7.214521000	-1.944223000	21.963063000
H	7.575602000	-2.247935000	20.986971000
H	5.348947000	-3.147342000	22.077232000
C	6.007850000	-2.428546000	22.553463000
C	5.754487000	-1.907693000	23.797483000
S	7.023777000	-0.795190000	24.257558000
C	4.565222000	-2.166455000	24.687665000
H	4.087104000	-3.093441000	24.348638000
H	2.289870000	-1.929680000	23.233873000
C	2.805247000	-0.980136000	23.429173000
H	2.062263000	-0.188110000	23.518111000
H	3.443591000	-0.753440000	22.568491000
N	3.546486000	-1.103379000	24.675570000
H	8.689433000	3.961351000	19.107165000

C	8.086194000	4.597956000	19.739452000
C	8.389098000	5.102431000	20.972409000
H	9.326326000	4.910977000	21.482487000
H	7.382638000	6.395370000	22.477971000
C	7.337057000	5.904495000	21.511885000
C	6.245022000	6.005524000	20.691302000
S	6.506027000	5.094942000	19.219223000
C	4.987814000	6.789096000	20.929202000
H	5.153484000	7.424358000	21.810663000
H	2.524761000	7.316442000	20.095965000
C	2.541922000	6.732637000	21.023149000
H	1.685880000	6.052217000	21.010845000
H	2.414476000	7.422198000	21.869699000
N	3.781918000	5.948340000	21.079568000
H	5.698697000	-0.678794000	28.751636000
H	4.877070000	-2.315182000	25.725099000
H	4.796927000	7.456139000	20.078970000
H	4.725236000	1.245498000	20.042104000
H	3.309009000	0.746751000	20.989362000
H	4.798296000	1.216560000	21.822334000
Mg	4.196661000	4.149690000	26.302916000

Conformer V

153

N	1.564500000	1.245690000	-0.123915000
C	1.490986000	2.615861000	-0.125503000
C	2.840684000	3.173238000	-0.104670000
C	3.713579000	2.088583000	0.007280000
C	2.875326000	0.875526000	-0.046365000
N	3.362223000	-0.372280000	-0.076807000
H	7.888092000	0.538861000	-4.623303000
C	7.212600000	1.100642000	-3.993173000
C	5.956400000	1.558728000	-4.282067000
H	5.469391000	1.396175000	-5.237091000
H	4.378920000	2.729957000	-3.244043000
C	5.364970000	2.281155000	-3.200496000
C	6.169828000	2.369316000	-2.091810000
S	7.689115000	1.545783000	-2.384104000
C	5.855100000	3.012457000	-0.761482000
H	5.269515000	3.919403000	-0.930970000
H	6.778890000	3.301257000	-0.249031000
H	6.700082000	1.542065000	1.314201000
C	5.842115000	1.090188000	0.801645000
H	6.221628000	0.336350000	0.102390000
N	5.086978000	2.148968000	0.148913000
H	3.910379000	5.499236000	-5.650461000
C	3.361054000	5.333079000	-4.734110000
C	2.182384000	4.662703000	-4.556251000
H	1.635483000	4.193792000	-5.366442000
H	0.835987000	4.185362000	-2.848030000
C	1.749659000	4.656475000	-3.192761000

C	2.599670000	5.316014000	-2.342489000
S	3.961086000	5.966150000	-3.232889000
C	2.468906000	5.508137000	-0.855864000
H	2.857132000	6.492717000	-0.578018000
H	2.818316000	5.143798000	1.950908000
C	3.659097000	5.002518000	1.252871000
H	4.351534000	4.278362000	1.688575000
H	4.184034000	5.957024000	1.146855000
N	3.231352000	4.525829000	-0.065986000
H	5.217790000	0.584907000	1.537249000
H	1.403680000	5.495571000	-0.606179000
N	1.252352000	-1.556195000	-0.097758000
C	2.614544000	-1.482783000	-0.116422000
C	3.168812000	-2.850025000	-0.075629000
C	2.076730000	-3.710341000	0.053889000
C	0.881511000	-2.874583000	-0.016025000
N	-0.371562000	-3.351800000	-0.059916000
N	-1.564507000	-1.245738000	-0.123925000
C	-1.490991000	-2.615909000	-0.125387000
C	-2.840692000	-3.173283000	-0.104624000
C	-3.713597000	-2.088618000	0.007152000
C	-2.875340000	-0.875568000	-0.046521000
N	-3.362232000	0.372238000	-0.077111000
H	-7.887646000	-0.539219000	-4.623949000
C	-7.212218000	-1.100957000	-3.993711000
C	-5.955991000	-1.559067000	-4.282448000
H	-5.468887000	-1.396584000	-5.237436000
H	-4.378618000	-2.730228000	-3.244185000
C	-5.364670000	-2.281419000	-3.200767000
C	-6.169638000	-2.369497000	-2.092153000
S	-7.688893000	-1.545980000	-2.384658000
C	-5.855044000	-3.012543000	-0.761748000
H	-5.269445000	-3.919505000	-0.931111000
H	-6.778885000	-3.301304000	-0.249368000
H	-6.700222000	-1.541989000	1.313749000
C	-5.842208000	-1.090157000	0.801235000
H	-6.221652000	-0.336377000	0.101883000
N	-5.087010000	-2.148992000	0.148663000
H	-3.909807000	-5.499718000	-5.650326000
C	-3.360577000	-5.333495000	-4.733929000
C	-2.181914000	-4.663124000	-4.555999000
H	-1.634921000	-4.194288000	-5.366170000
H	-0.835693000	-4.185666000	-2.847673000
C	-1.749335000	-4.656792000	-3.192463000
C	-2.599447000	-5.316249000	-2.342229000
S	-3.960780000	-5.966435000	-3.232721000
C	-2.468848000	-5.508252000	-0.855574000
H	-2.857115000	-6.492803000	-0.577690000
H	-2.818543000	-5.143665000	1.951130000
C	-3.659252000	-5.002442000	1.252994000
H	-4.351730000	-4.278246000	1.688563000
H	-4.184181000	-5.956956000	1.147007000
N	-3.231367000	-4.525869000	-0.065860000
H	-5.217949000	-0.584815000	1.536856000

H	-1.403647000	-5.495678000	-0.605776000
N	-1.252362000	1.556151000	-0.098007000
C	-2.614553000	1.482736000	-0.116762000
C	-3.168823000	2.849984000	-0.076122000
C	-2.076749000	3.710310000	0.053395000
C	-0.881526000	2.874545000	-0.016360000
N	0.371550000	3.351758000	-0.060192000
H	3.342385000	-5.234924000	-1.616590000
C	2.500340000	-5.759893000	-1.159340000
H	1.660934000	-5.759956000	-1.873338000
H	2.800630000	-6.796932000	-0.979521000
N	2.166168000	-5.115566000	0.114495000
C	1.190634000	-5.853466000	0.935327000
H	0.159472000	-5.635341000	0.641252000
H	1.367447000	-6.915620000	0.740248000
C	1.320991000	-5.584002000	2.409827000
S	2.487150000	-6.442243000	3.395896000
C	2.000456000	-5.586577000	4.826093000
C	0.999260000	-4.694916000	4.556315000
C	0.612631000	-4.696874000	3.179054000
H	-0.167579000	-4.066901000	2.766855000
H	0.546426000	-4.061288000	5.310535000
H	2.477594000	-5.798137000	5.773002000
H	4.998736000	-1.805625000	-1.618625000
C	5.482897000	-2.385341000	-0.833647000
H	6.232003000	-3.043821000	-1.290198000
H	6.002613000	-1.688115000	-0.167091000
N	4.500063000	-3.210612000	-0.147958000
C	5.036241000	-4.157241000	0.841560000
H	4.260127000	-4.899584000	1.043494000
H	5.885017000	-4.676831000	0.384203000
H	3.595158000	-3.333794000	3.223426000
C	4.661614000	-3.139505000	3.197548000
C	5.455273000	-3.506576000	2.139194000
S	7.118769000	-3.049506000	2.447207000
C	6.714820000	-2.374988000	3.995103000
C	5.376646000	-2.494571000	4.253146000
H	4.914206000	-2.143821000	5.169004000
H	7.486476000	-1.942784000	4.616939000
H	-3.342244000	5.234743000	-1.617342000
C	-2.500238000	5.759750000	-1.160064000
H	-2.800540000	6.796807000	-0.980372000
H	-1.660767000	5.759740000	-1.873985000
N	-2.166187000	5.115542000	0.113864000
C	-1.190715000	5.853513000	0.934707000
H	-1.367493000	6.915649000	0.739503000
H	-0.159529000	5.635341000	0.640744000
C	-1.321205000	5.584204000	2.409224000
S	-2.487413000	6.442591000	3.395108000
C	-2.000870000	5.587055000	4.825433000
H	-2.478079000	5.798733000	5.772280000
C	-0.999686000	4.695329000	4.555831000
H	-0.546939000	4.061762000	5.310154000
C	-0.612943000	4.697129000	3.178601000

H	0.167279000	4.067085000	2.766533000
H	-4.998656000	1.805394000	-1.619095000
H	-6.002660000	1.688108000	-0.167631000
C	-5.482867000	2.385228000	-0.834237000
H	-6.231916000	3.043670000	-1.290938000
N	-4.500069000	3.210560000	-0.148571000
C	-5.036308000	4.157322000	0.840786000
H	-5.885040000	4.676868000	0.383299000
H	-4.260198000	4.899677000	1.042689000
C	-5.455452000	3.506826000	2.138469000
S	-7.118978000	3.049810000	2.446404000
C	-6.715162000	2.375497000	3.994424000
C	-5.377010000	2.495104000	4.252563000
C	-4.661884000	3.139889000	3.196939000
H	-3.595429000	3.334174000	3.222880000
H	-4.914649000	2.144473000	5.168506000
H	-7.486875000	1.943382000	4.616254000
Mg	0.000002000	-0.000029000	-0.281726000

Conformer VI

153

N	-1.797377000	0.879716000	-0.009429000
C	-3.001149000	0.224105000	-0.065953000
C	-4.082868000	1.203194000	-0.103402000
C	-3.484802000	2.458919000	0.028037000
C	-2.026733000	2.223283000	0.037057000
N	-1.108016000	3.198418000	0.010786000
H	-3.800420000	6.980640000	-4.583837000
C	-4.007077000	6.115317000	-3.969456000
C	-3.801732000	4.793835000	-4.257922000
H	-3.379729000	4.448589000	-5.195152000
H	-4.139383000	2.842571000	-3.249557000
C	-4.213213000	3.923322000	-3.202370000
C	-4.727789000	4.583028000	-2.113923000
S	-4.706225000	6.312825000	-2.392737000
C	-5.212839000	3.993938000	-0.809833000
H	-5.759625000	3.069698000	-1.011411000
H	-5.899692000	4.686861000	-0.312090000
H	-4.324516000	5.387664000	1.298980000
C	-3.528116000	4.810931000	0.812889000
H	-2.987113000	5.481012000	0.135394000
N	-4.131246000	3.674389000	0.133757000
H	-6.316729000	1.202395000	-5.770222000
C	-5.988637000	0.767699000	-4.836163000
C	-4.900389000	-0.026466000	-4.601113000
H	-4.202489000	-0.325339000	-5.375073000
H	-4.001610000	-1.058791000	-2.844869000
C	-4.790519000	-0.423206000	-3.231159000
C	-5.789556000	0.071897000	-2.432943000
S	-6.895599000	1.044157000	-3.381711000
C	-5.991161000	-0.132498000	-0.956024000

H	-7.061407000	-0.201301000	-0.738087000
H	-5.940670000	0.313583000	1.859025000
C	-6.146523000	1.139277000	1.158893000
H	-5.814742000	2.072930000	1.619440000
H	-7.228500000	1.199464000	1.004948000
N	-5.473116000	0.974313000	-0.132974000
H	-2.830284000	4.462891000	1.573242000
H	-5.541100000	-1.092093000	-0.683470000
N	0.869065000	1.803575000	0.014802000
C	0.217324000	3.001497000	-0.023177000
C	1.216460000	4.088233000	-0.023381000
C	2.465489000	3.472141000	0.084818000
C	2.221040000	2.032817000	0.052042000
N	3.186531000	1.103599000	-0.012273000
N	1.797377000	-0.879716000	-0.009429000
C	3.001149000	-0.224105000	-0.065953000
C	4.082868000	-1.203194000	-0.103402000
C	3.484802000	-2.458919000	0.028037000
C	2.026733000	-2.223283000	0.037057000
N	1.108016000	-3.198418000	0.010786000
H	3.800420000	-6.980640000	-4.583837000
C	4.007077000	-6.115317000	-3.969456000
C	3.801732000	-4.793835000	-4.257922000
H	3.379729000	-4.448589000	-5.195152000
H	4.139383000	-2.842571000	-3.249557000
C	4.213213000	-3.923322000	-3.202370000
C	4.727789000	-4.583028000	-2.113923000
S	4.706225000	-6.312825000	-2.392737000
C	5.212839000	-3.993938000	-0.809833000
H	5.759625000	-3.069698000	-1.011411000
H	5.899692000	-4.686861000	-0.312090000
H	4.324516000	-5.387664000	1.298980000
C	3.528116000	-4.810931000	0.812889000
H	2.987113000	-5.481012000	0.135394000
N	4.131246000	-3.674389000	0.133757000
H	6.316729000	-1.202395000	-5.770222000
C	5.988637000	-0.767699000	-4.836163000
C	4.900389000	0.026466000	-4.601113000
H	4.202489000	0.325339000	-5.375073000
H	4.001610000	1.058791000	-2.844869000
C	4.790519000	0.423206000	-3.231159000
C	5.789556000	-0.071897000	-2.432943000
S	6.895599000	-1.044157000	-3.381711000
C	5.991161000	0.132498000	-0.956024000
H	7.061407000	0.201301000	-0.738087000
H	5.940670000	-0.313583000	1.859025000
C	6.146523000	-1.139277000	1.158893000
H	5.814742000	-2.072930000	1.619440000
H	7.228500000	-1.199464000	1.004948000
N	5.473116000	-0.974313000	-0.132974000
H	2.830284000	-4.462891000	1.573242000
H	5.541100000	1.092093000	-0.683470000
N	-0.869065000	-1.803575000	0.014802000
C	-0.217324000	-3.001497000	-0.023177000

C	-1.216460000	-4.088233000	-0.023381000
C	-2.465489000	-3.472141000	0.084818000
C	-2.221040000	-2.032817000	0.052042000
N	-3.186531000	-1.103599000	-0.012273000
H	3.233964000	5.262592000	-1.624576000
C	4.086529000	4.728551000	-1.198306000
H	4.418165000	3.967327000	-1.922660000
H	4.900932000	5.444994000	-1.052460000
N	3.697870000	4.154326000	0.093595000
C	4.814045000	3.596268000	0.876454000
H	5.045401000	2.567883000	0.583570000
H	5.690277000	4.207525000	0.639447000
C	4.573739000	3.613340000	2.361599000
S	4.865268000	5.051166000	3.319108000
C	4.373060000	4.252930000	4.780328000
C	4.007077000	2.957216000	4.541018000
C	4.124323000	2.594748000	3.162176000
H	3.888923000	1.611529000	2.770564000
H	3.670722000	2.279846000	5.317825000
H	4.389068000	4.786169000	5.720820000
H	-0.560474000	5.280188000	-1.530786000
C	-0.217324000	5.972162000	-0.762872000
H	0.038545000	6.928314000	-1.235416000
H	-1.045566000	6.146671000	-0.066853000
N	0.974122000	5.445593000	-0.114857000
C	1.632646000	6.349739000	0.840824000
H	2.642069000	5.968884000	1.014603000
H	1.722670000	7.332073000	0.365162000
H	1.582697000	4.727715000	3.247729000
C	0.954461000	5.611262000	3.232893000
C	0.911473000	6.470608000	2.163090000
S	-0.195928000	7.788040000	2.493620000
C	-0.578076000	7.158269000	4.065689000
C	0.106775000	6.000540000	4.315062000
H	0.018789000	5.445896000	5.242596000
H	-1.273409000	7.682857000	4.706235000
H	-3.233964000	-5.262592000	-1.624576000
C	-4.086529000	-4.728551000	-1.198306000
H	-4.900932000	-5.444994000	-1.052460000
H	-4.418165000	-3.967327000	-1.922660000
N	-3.697870000	-4.154326000	0.093595000
C	-4.814045000	-3.596268000	0.876454000
H	-5.690277000	-4.207525000	0.639447000
H	-5.045401000	-2.567883000	0.583570000
C	-4.573739000	-3.613340000	2.361599000
S	-4.865268000	-5.051166000	3.319108000
C	-4.373060000	-4.252930000	4.780328000
H	-4.389068000	-4.786169000	5.720820000
C	-4.007077000	-2.957216000	4.541018000
H	-3.670722000	-2.279846000	5.317825000
C	-4.124323000	-2.594748000	3.162176000
H	-3.888923000	-1.611529000	2.770564000
H	0.560474000	-5.280188000	-1.530786000
H	1.045566000	-6.146671000	-0.066853000

C	0.217324000	-5.972162000	-0.762872000
H	-0.038545000	-6.928314000	-1.235416000
N	-0.974122000	-5.445593000	-0.114857000
C	-1.632646000	-6.349739000	0.840824000
H	-1.722670000	-7.332073000	0.365162000
H	-2.642069000	-5.968884000	1.014603000
C	-0.911473000	-6.470608000	2.163090000
S	0.195928000	-7.788040000	2.493620000
C	0.578076000	-7.158269000	4.065689000
C	-0.106775000	-6.000540000	4.315062000
C	-0.954461000	-5.611262000	3.232893000
H	-1.582697000	-4.727715000	3.247729000
H	-0.018789000	-5.445896000	5.242596000
H	1.273409000	-7.682857000	4.706235000
Mg	0.000000000	0.000000000	0.008563000

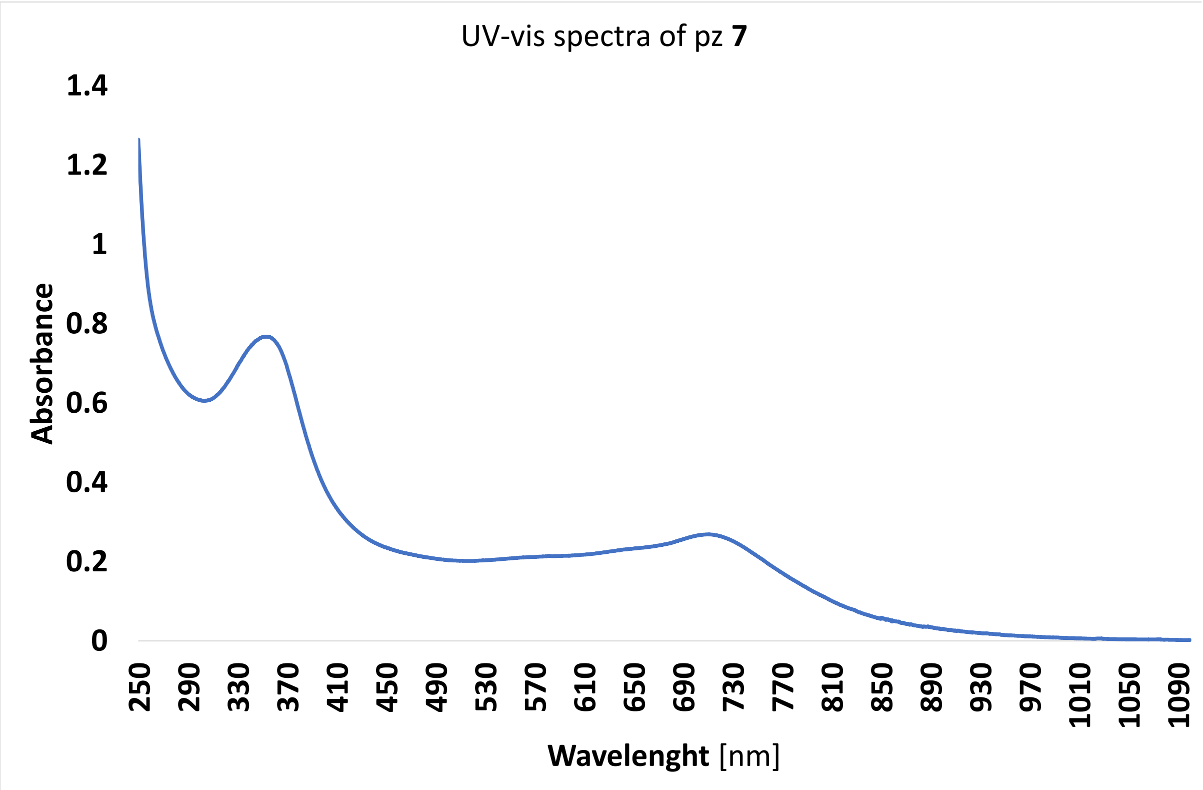

UV-vis spectra of pz **7** in DCM
